# Synthesis and characterization data of monocationic and dicationic ionic liquids or molten salts

**DOI:** 10.1016/j.dib.2018.05.080

**Published:** 2018-05-24

**Authors:** M.G. Montalbán, G. Víllora, P. Licence

**Affiliations:** a Department of Chemical Engineering, Faculty of Chemistry, Regional Campus of International Excellence "Campus Mare Nostrum", University of Murcia, P.O. Box 4021, Campus of Espinardo, E-30071 Murcia, Spain; b School of Chemistry, The University of Nottingham, Nottingham NG7 2RD, UK

## Abstract

Data presented in this article are related with the research paper entitled “Ecotoxicity assessment of dicationic *versus* monocationic ionic liquids as a more environmentally friendly alternative” [1]. The present article describes the synthesis steps and characterization data of a set of twenty-six imidazolium, pyrrolidinium and pyridinium-based ionic liquids (ILs) or molten salts: nine monocationic and seventeen dicationic. Specifically, the chemical structure of the compounds was confirmed by ^1^H NMR, ^13^C NMR and ^19^F NMR spectroscopy and mass spectrometry (MS). Other data such as physical state at room temperature, melting point temperature (for solids at room temperature) and thermal decomposition temperature (when melting was not reached before decomposition) of the ILs or molten salts are also reported here.

**Specifications Table**

**Table:** 

Subject area	*Chemistry*
More specific subject area	*Synthesis and characterization of ionic liquids or molten salts*
Type of data	*Tables, figures*
How data was acquired	^1^H, ^13^C and ^19^F NMR spectra were recorded on a Jeol model EX270 instrument.
	MS was recorded on a Bruker MicroTOF 61 spectrometer.
	Melting point temperature was recorded on a Büchi Melting Point B-540 instrument.
	Thermal decomposition temperature was acquired by Differential Scanning Calorimetry on a DSC 2920 (TA instruments) instrument.
Data format	*Raw and analyzed data*
Experimental factors	*Samples were dried under vacuum overnight before their characterization.*
Experimental features	*Relevant data on the characterization of the ILs or molten salts were determined.*
Data source location	*University of Murcia, Murcia, Spain, Europe*
Data accessibility	*The data are provided with this article.*
Related research article	*M.G. Montalbán, G. Víllora, P. Licence, Ecotoxicity assessment of dicationic versus monocationic ionic liquids as a more environmentally friendly alternative, Ecotox. Environ. Safe. 150 (2018) 129–135.*

**Value of the Data**

• All the steps for the synthesis of the ILs or molten salts here described and the methods can be followed by other researchers.
• The chemical synthesis of some of these ILs or molten salts had not been reported before.
• NMR spectra and MS data of the ILs or molten salts synthesized are useful for structural characterization of these and other similar ILs or molten salts.
• Data on melting point and decomposition temperature of these ILs or molten salts can be valuable for the design of their applications.

## 1 Data

**Fig. 1:**
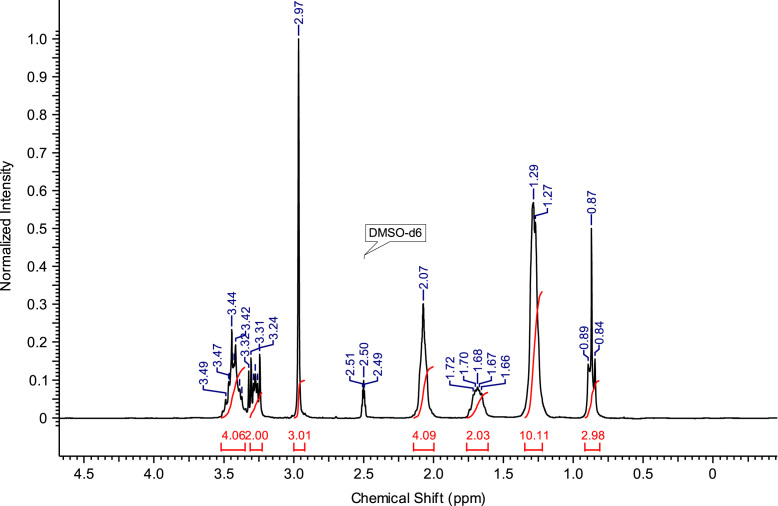
^1^H NMR spectra of C_8_(MPyrr)SbF_6_.

**Fig. 2:**
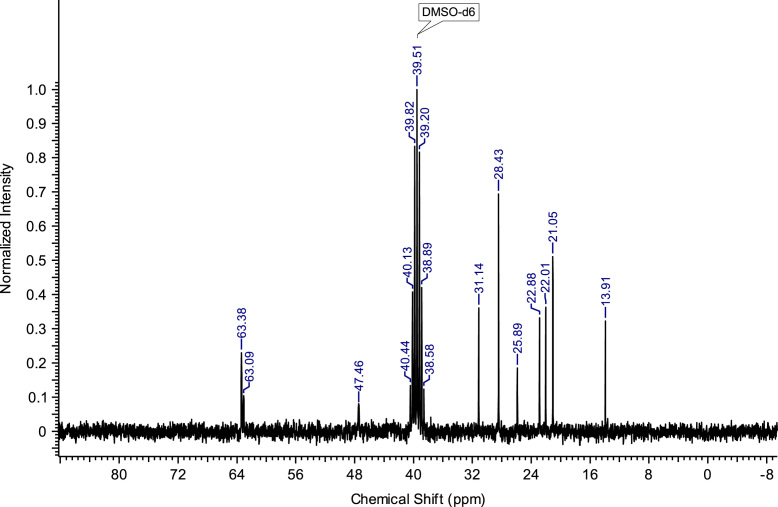
^13^C NMR spectra of C_8_(MPyrr)SbF_6_.

**Fig. 3:**
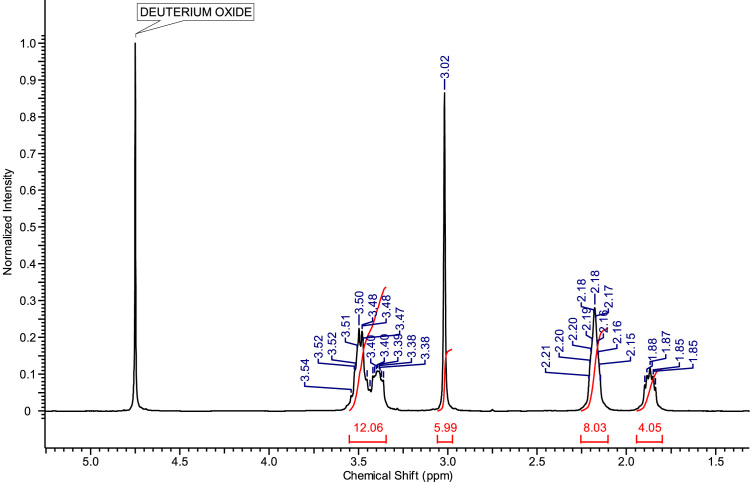
^1^H NMR spectra of C_4_(MPyrr)_2_Br_2_.

**Fig. 4:**
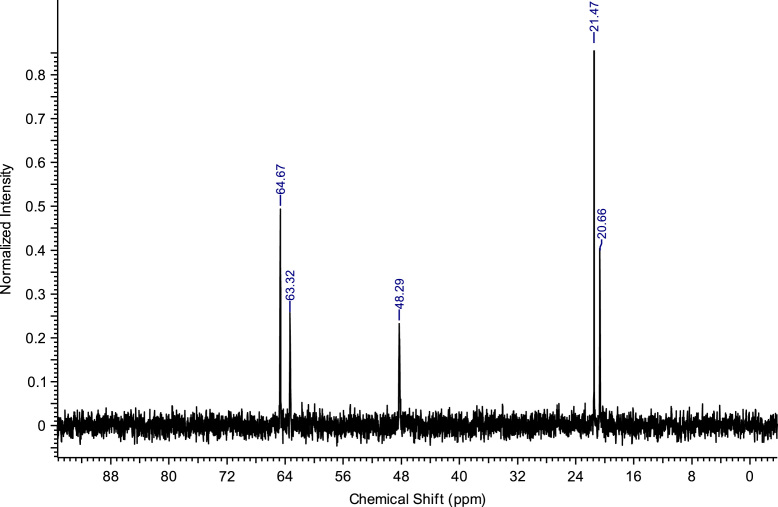
^13^C NMR spectra of C_4_(MPyrr)_2_Br_2_.

**Fig. 5:**
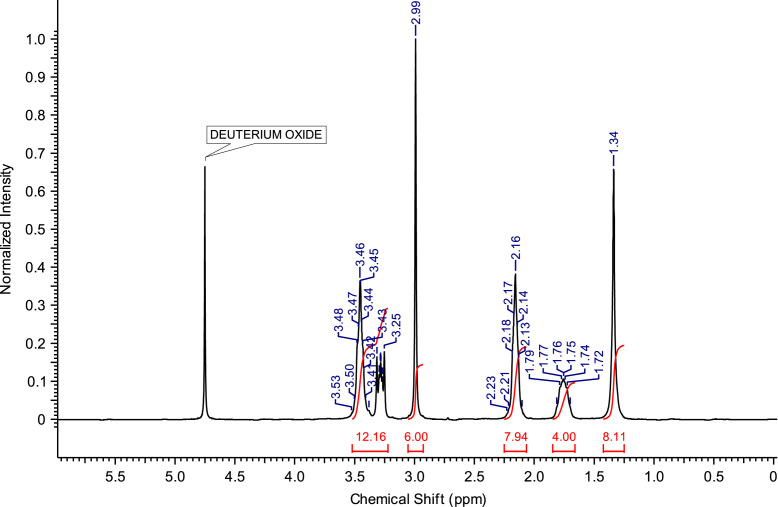
^1^H NMR spectra of C_8_(MPyrr)_2_Br_2_.

**Fig. 6:**
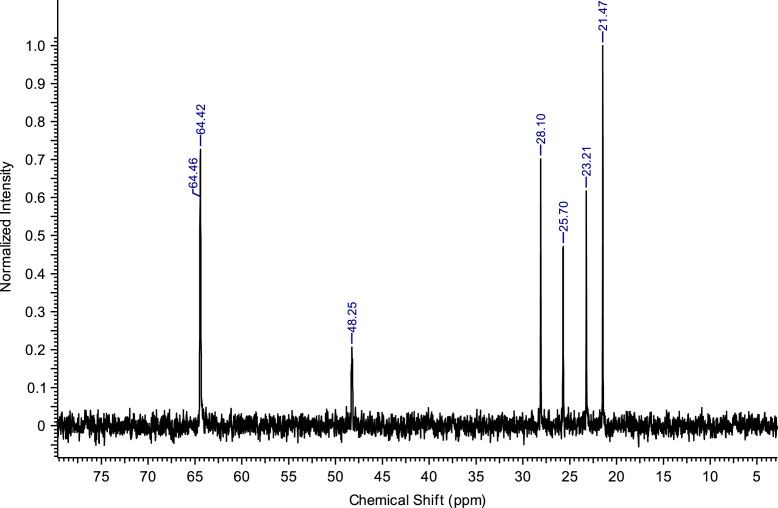
^13^C NMR spectra of C_8_(MPyrr)_2_Br_2_.

**Fig. 7:**
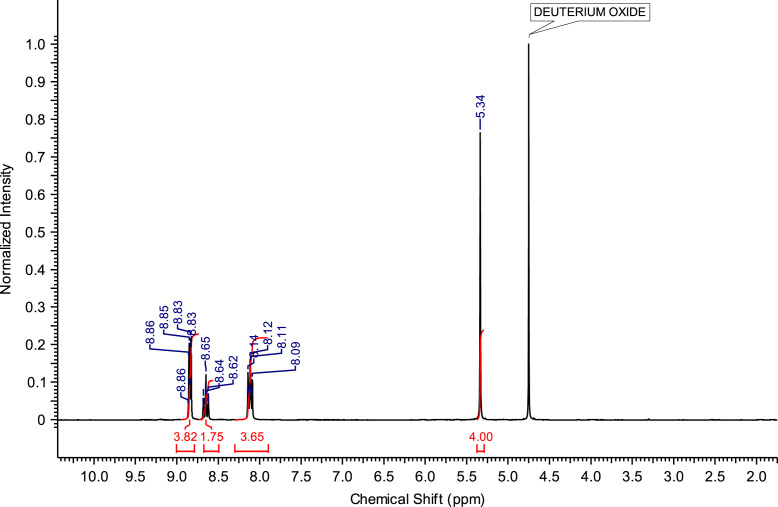
^1^H NMR spectra of C_2_(Pyr)_2_Br_2_.

**Fig. 8:**
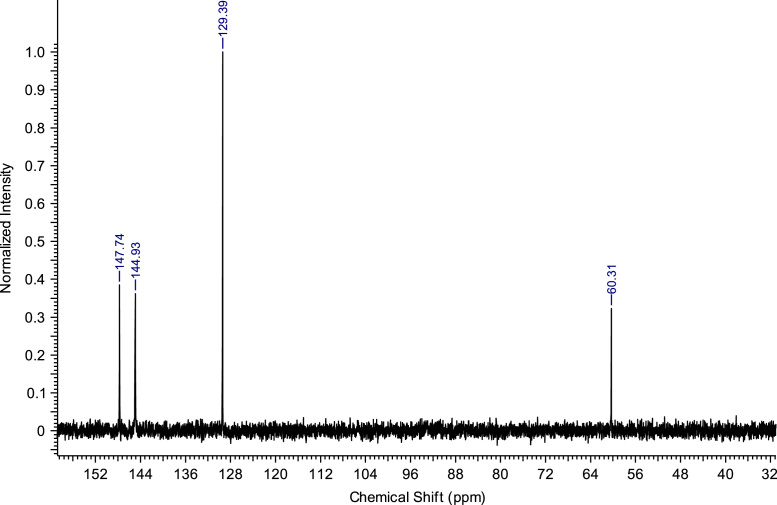
^13^C NMR spectra of C_2_(Pyr)_2_Br_2_.

**Fig. 9:**
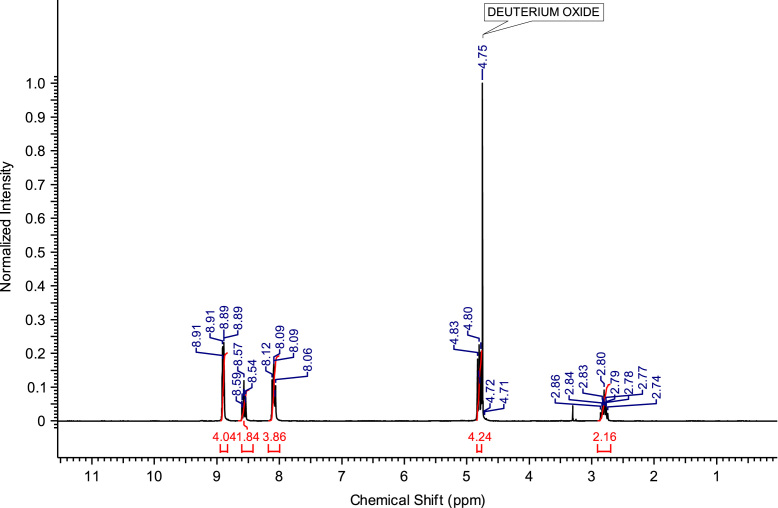
^1^H NMR spectra of C_3_(Pyr)_2_Br_2_.

**Fig. 10:**
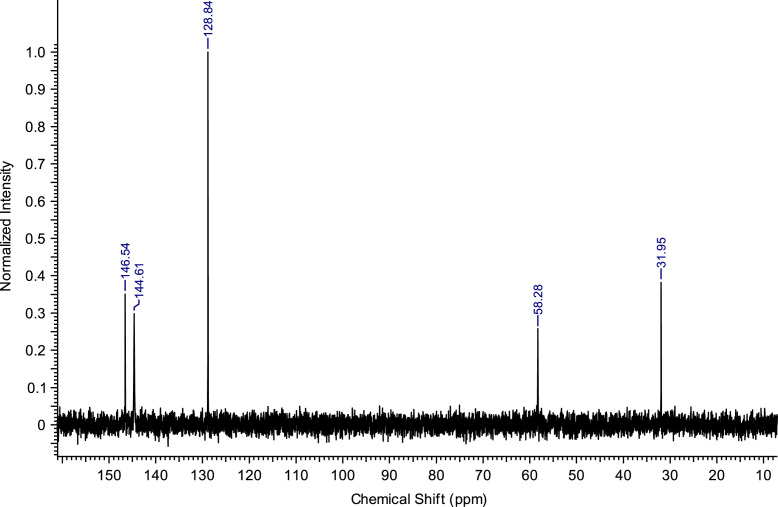
^13^C NMR spectra of C_3_(Pyr)_2_Br_2_.

**Fig. 11:**
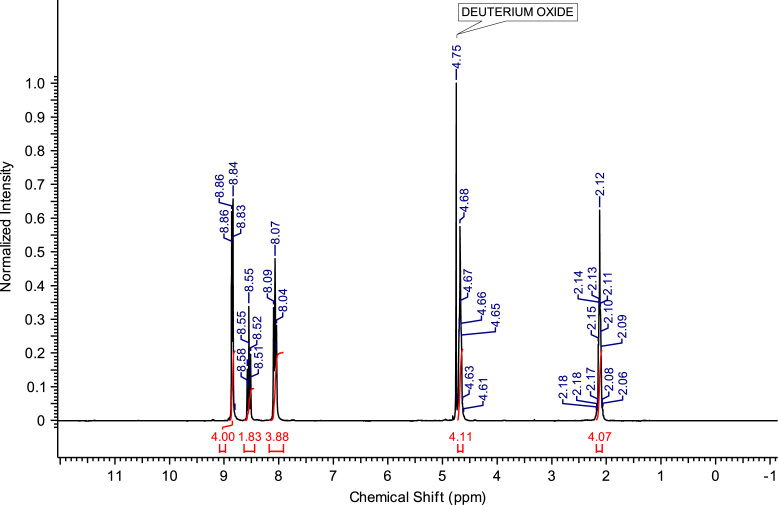
^1^H NMR spectra of C_4_(Pyr)_2_Br_2_.

**Fig. 12:**
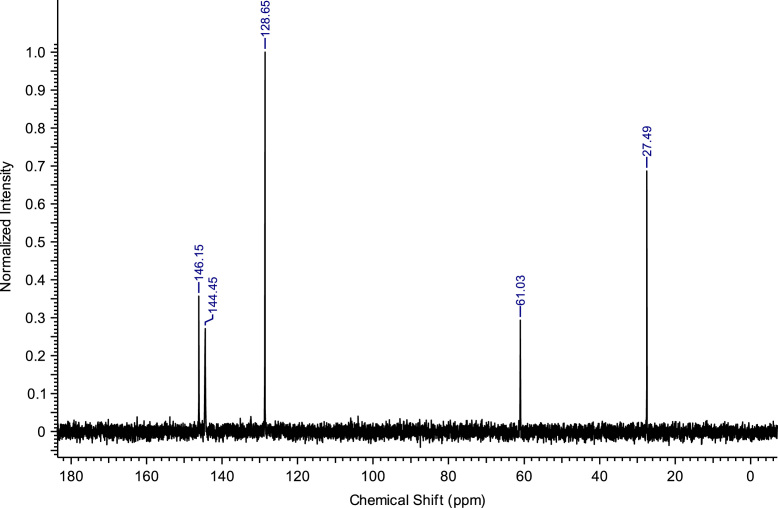
^13^C NMR spectra of C_4_(Pyr)_2_Br_2_.

**Fig. 13:**
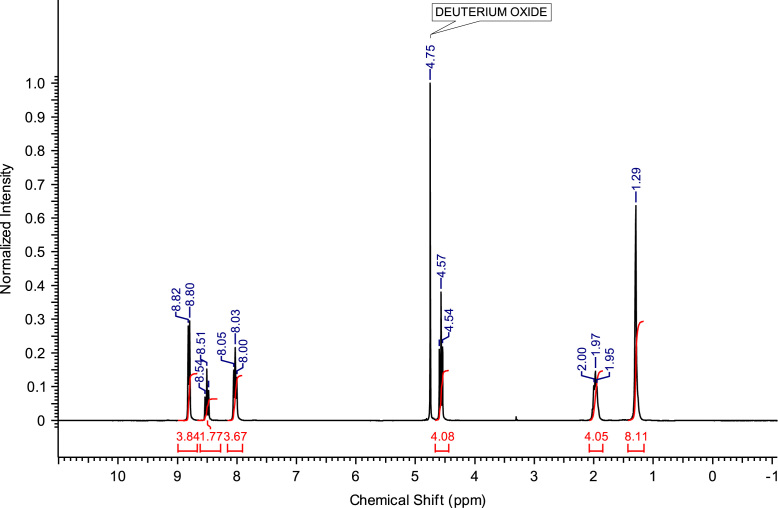
^1^H NMR spectra of C_8_(Pyr)_2_Br_2_.

**Fig. 14:**
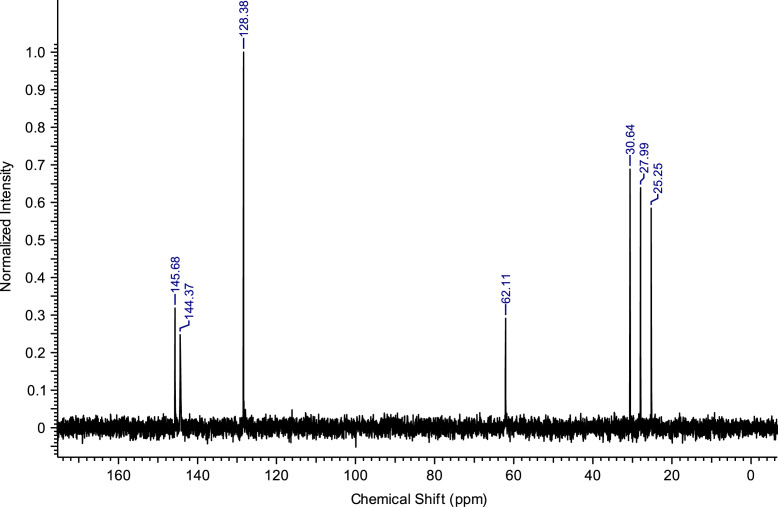
^13^C NMR spectra of C_8_(Pyr)_2_Br_2_.

**Fig. 15:**
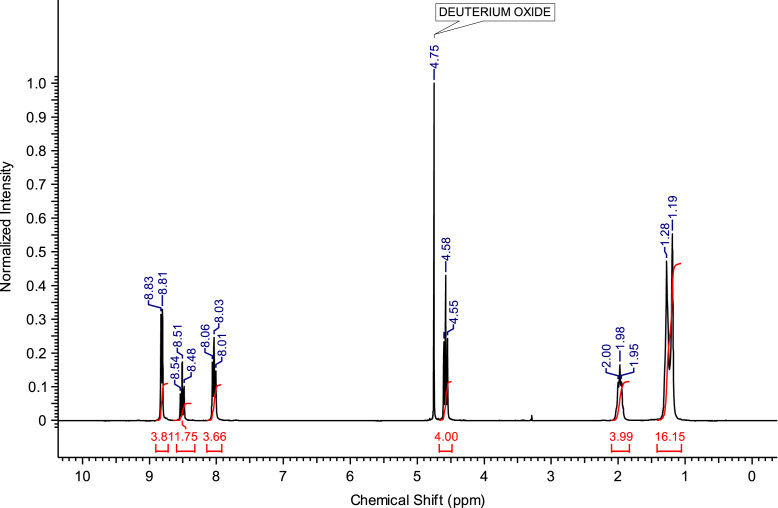
^1^H NMR spectra of C_12_(Pyr)_2_Br_2_.

**Fig. 16:**
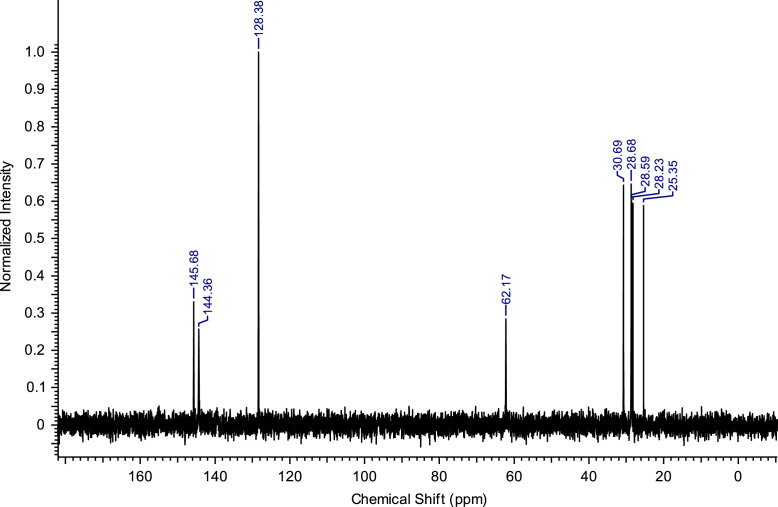
^13^C NMR spectra of C_12_(Pyr)_2_Br_2_.

**Fig. 17:**
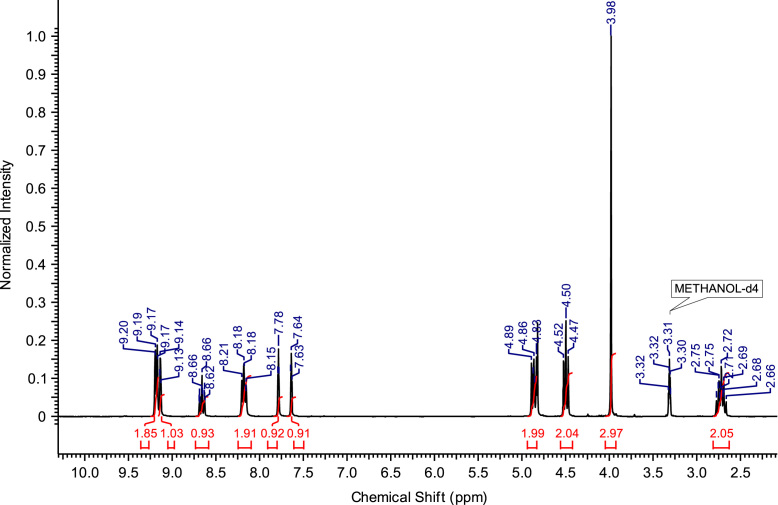
^1^H NMR spectra of C_3_(Pyr)(MIm)Br_2_.

**Fig. 18:**
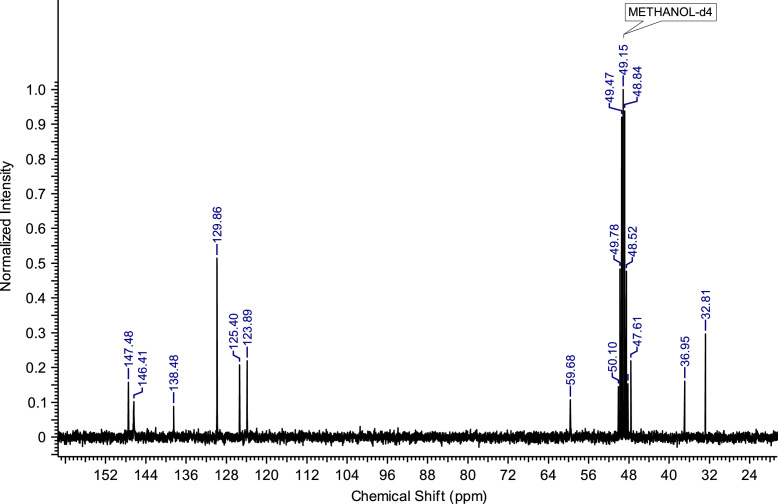
^13^C NMR spectra of C_3_(Pyr)(MIm)Br_2_.

**Fig. 19:**
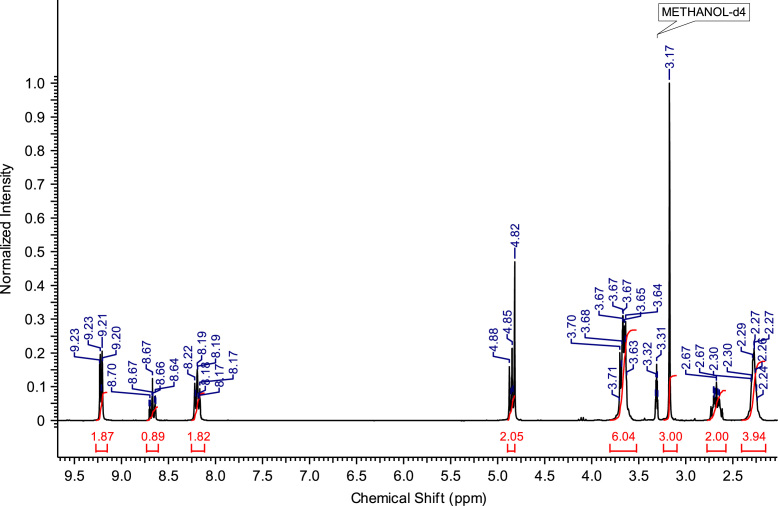
^1^H NMR spectra of C_3_(Pyr)(MPyrr)Br_2_.

**Fig. 20:**
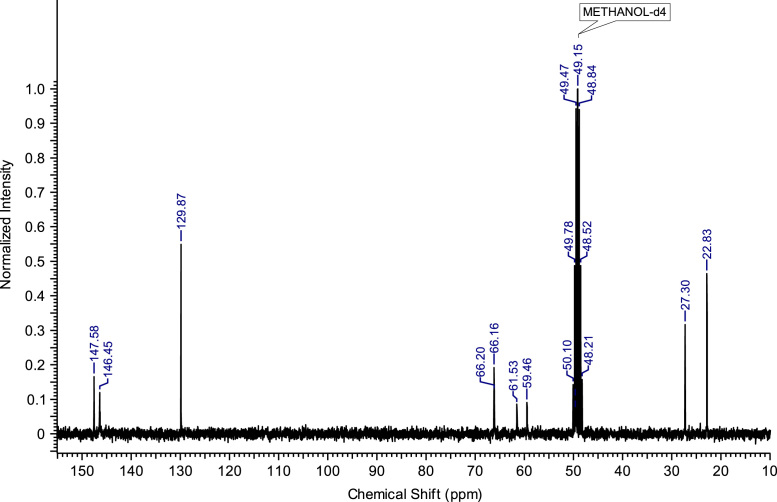
^13^C NMR spectra of C_3_(Pyr)(MPyrr)Br_2_.

The abbreviations, molecular weights and structures of the ILs or molten salts are presented in Table 1. The synthesis steps necessary for the preparation of the ILs or molten salts are described then in detail. After the report of the chemical synthesis of the ILs or molten salts, their characterization (NMR spectra and MS) is included. Fig. 1, Fig. 2, Fig. 3, Fig. 4, Fig. 5, Fig. 6, Fig. 7, Fig. 8, Fig. 9, Fig. 10, Fig. 11, Fig. 12, Fig. 13, Fig. 14, Fig. 15, Fig. 16, Fig. 17, Fig. 18, Fig. 19, Fig. 20 show the ^1^H NMR and ^13^C NMR spectra of the novel compounds. Finally, Table 2 collects the physical state of the ILs or molten salts at room temperature, their colour, melting point (for solids at room temperature) and decomposition temperature (when melting is not reached before thermal decomposition).

**Table 1.** Abbreviations and structures of the studied ILs or molten salts.

**Abbreviation**	**Molecular weight (g mol**^**−1**^**)**	**Structure**
**C**_**8**_**(MIm) Br**	275.23	
**C**_**8**_**(MIm) NTf**_**2**_	475.47	
**C**_**8**_**(MIm) SbF**_**6**_	431.07	
**C**_**8**_**(Pyr) Br**	272.22	
**C**_**8**_**(Pyr) NTf**_**2**_	472.47	
**C**_**8**_**(Pyr) SbF**_**6**_	428.07	
**C**_**8**_**(MPyrr) Br**	278.27	
**C**_**8**_**(MPyrr) NTf**_**2**_	478.51	
**C**_**8**_**(MPyrr) SbF**_**6**_	434.12	
**C**_**2**_**(MIm)**_**2**_**Br**_**2**_	352.07	
**C**_**3**_**(MIm)**_**2**_**Br**_**2**_	366.07	
**C**_**4**_**(MIm)**_**2**_**Br**_**2**_	380.12	
**C**_**6**_**(MIm)**_**2**_**Br**_**2**_	408.18	
**C**_**8**_**(MIm)**_**2**_**Br**_**2**_	436.23	
**C**_**3**_**(MPyrr)**_**2**_**Br**_**2**_	372.21	
**C**_**4**_**(MPyrr)**_**2**_**Br**_**2**_	386.21	
**C**_**6**_**(MPyrr)**_**2**_**Br**_**2**_	414.26	
**C**_**8**_**(MPyrr)**_**2**_**Br**_**2**_	442.32	
**C**_**2**_**(Pyr)**_**2**_**Br**_**2**_	346.06	
**C**_**3**_**(Pyr)**_**2**_**Br**_**2**_	360.09	
**C**_**4**_**(Pyr)**_**2**_**Br**_**2**_	374.09	
**C**_**6**_**(Pyr)**_**2**_**Br**_**2**_	402.09	
**C**_**8**_**(Pyr)**_**2**_**Br**_**2**_	430.09	
**C**_**12**_**(Pyr)**_**2**_**Br**_**2**_	486.09	
**C**_**3**_**(Pyr)(MIm) Br**_**2**_	363.12	
**C**_**3**_**(Pyr)(MPyrr) Br**_**2**_	366.14

**Table 2:** Physical state, colour and melting point or thermal decomposition temperature of the synthesized ILs or molten salts.

**Ionic liquid/Molten salt**	**Physical state at rt**^a^	**Colour**	**Melting point temperature (°C)**	**Thermal decomposition temperature**^b^**(°C)**
**C**_**8**_**(MIm) Br**	Liquid	Colourless	–	–
**C**_**8**_**(MIm) NTf**_**2**_	Liquid	Orange	–	–
**C**_**8**_**(MIm) SbF**_**6**_	Liquid	Light yellow	–	–
**C**_**8**_**(Pyr) Br**	Solid	White	46	–
**C**_**8**_**(Pyr) NTf**_**2**_	Liquid	Light yellow	–	–
**C**_**8**_**(Pyr) SbF**_**6**_	Solid	White	82	–
**C**_**8**_**(MPyrr) Br**	Solid	Light yellow	151	–
**C**_**8**_**(MPyrr) NTf**_**2**_	Liquid	Light yellow	–	–
**C**_**8**_**(MPyrr) SbF**_**6**_	Solid	White	46	–
**C**_**2**_**(MIm)**_**2**_**Br**_**2**_	Solid	White	229	–
**C**_**3**_**(MIm)**_**2**_**Br**_**2**_	Solid	White	132	–
**C**_**4**_**(MIm)**_**2**_**Br**_**2**_	Liquid	Colourless	–	–
**C**_**6**_**(MIm)**_**2**_**Br**_**2**_	Solid	White	156	–
**C**_**8**_**(MIm)**_**2**_**Br**_**2**_	Solid	White	75	–
**C**_**3**_**(MPyrr)**_**2**_**Br**_**2**_	Liquid	Orange	–	–
**C**_**4**_**(MPyrr)**_**2**_**Br**_**2**_	Solid	Light brown	–	314
**C**_**6**_**(MPyrr)**_**2**_**Br**_**2**_	Solid	White	173	–
**C**_**8**_**(MPyrr)**_**2**_**Br**_**2**_	Solid	White	181	–
**C**_**2**_**(Pyr)**_**2**_**Br**_**2**_	Solid	White	–	300
**C**_**3**_**(Pyr)**_**2**_**Br**_**2**_	Solid	White	241	–
**C**_**4**_**(Pyr)**_**2**_**Br**_**2**_	Solid	White	–	262
**C**_**6**_**(Pyr)**_**2**_**Br**_**2**_	Solid	White	–	265
**C**_**8**_**(Pyr)**_**2**_**Br**_**2**_	Solid	White	193	–
**C**_**12**_**(Pyr)**_**2**_**Br**_**2**_	Solid	White	140	–
**C**_**3**_**(Pyr)(MIm) Br**_**2**_	Solid	White	104	–
**C**_**3**_**(Pyr)(MPyrr) Br**_**2**_	Solid	Light brown	–	231

a Room temperature.

b Decomposition temperature is reached before the melting of the ionic liquid or the molten salt.

## 2 Experimental design, materials, and methods

See Table 1

## 3 Synthesis and characterization of ILs

**C**_**8**_**(MIm)Br** was synthesized following procedures described in the literature with modifications [2], [3]. 100 mmol of 1-methylimidazole was placed in a round bottom flask fitted with a reflux condenser and an additional funnel under a static atmosphere of Ar. 110 mmol of 1-bromooctane was added dropwise *via* a pressure equalising addition funnel while the mixture was stirred at 60 °C under inert atmosphere. The additional funnel was removed and the reaction mixture was stirred at 60 °C for 72 h. The crude product recovered as a honey coloured oil was dissolved in 20 mL of acetonitrile and washed with 250 mL of ethyl acetate to remove unreacted 1-bromooctane. The upper layer (ethyl acetate containing 1-bromooctane) was decanted set aside, residual acetonitrile was removed from the product by automated rotary evaporator. The oil was dried under high vacuum (*p* < 10^−2^ mbar) at 60 °C for 24 h and eventually recovered as a colourless liquid. Data in agreement with the literature [4].

**C**_**8**_**(MPyrr)Br** and **C**_**8**_**(Pyr)Br** were synthesized according to procedures described in the literature [5]. 240 mmol of 1-methylpyrrolidine or pyridine was placed in a round bottom flask fitted with a water condenser topped with a drying tube (CaCl_2_) to avoid moisture penetration. 288 mmol of 1-bromooctane was added dropwise while stirring at 70 °C. The reaction time was 48 hours. The desired product was recrystallised in acetonitrile/ethyl acetate (≈1:3 v:v) and dried first using a rotary evaporator and then under high vacuum (*p* < 10^−2^ mbar) at 60 °C for 12 h to yield a white solid. Data in agreement with the literature [6], [7].

**C**_**8**_**(MIm)NTf**_**2**_, **C**_**8**_**(MPyrr)NTf**_**2**_ and **C**_**8**_**(Pyr)NTf**_**2**_ were synthesized according to procedures described in the literature [5], [8], [9]. 5 mmol of C_8_(MIm)Br, C_8_(MPyrr)Br or C_8_(Pyr)Br were transferred to a round bottom flask and dissolved in 10 mL of ultrapure water while stirring at room temperature. Aqueous lithium bis(trifluoromethane)sulfonylimide (6 mmol in 6 mL of ultrapure water) was added dropwise. The mixture was stirred at room temperature for 24 hours. Then the mixture was transferred to a funnel washing with ethyl acetate; the aqueous layer was separated and the ionic liquid dissolved in 30 ml of ethyl acetate and washed with ultrapure water (4 × 30 mL). Finally, the ethyl acetate was removed in a rotary evaporator and the ionic liquid was dried under vacuum (*p* < 10^−2^ mbar) at 60 °C for 24 hours. Data in agreement with the literature [8], [10], [11].

**C**_**8**_**(MIm)SbF**_**6**_, **C**_**8**_**(MPyrr)SbF**_**6**_ and **C**_**8**_**(Pyr)SbF**_**6**_ were synthesized according to procedures described in the literature [12]. In a single necked round bottom flask with a magnetic stirring bar, 5 mmol of C_8_(MIm)Br, C_8_(MPyrr)Br or C_8_(Pyr)Br was dissolved in 20 mL of dichloromethane. Then 6 mmol of sodium hexafluoroantimonate (V) was added. The mixture was stirred for 24 hours at room temperature while observing the formation of a white solid (NaBr). This solid was filtered off and the filtrate was washed with ultrapure water several times (5 × 50 mL). The solvent was removed in a rotary evaporator and the ionic liquid was dried under high vacuum (*p* < 10^−2^ mbar) at 70 °C for 24 hours. Data in agreement with the literature [13].

**C**_**2**_ **(MIm)**_**2**_ **Br**_**2**_, **C**_**3**_ **(MIm)**_**2**_ **Br**_**2**_, **C**_**4**_ **(MIm)**_**2**_ **Br**_**2**_, **C**_**6**_ **(MIm)**_**2**_ **Br**_**2**_ and **C**_**8**_ **(MIm)**_**2**_ **Br**_**2**_ were synthesized following the same procedure [8], [14], [15]. A three-necked round bottom flask fitted with reflux condenser and pressure equation funnel was filled with a solution of 30 mmol of 1,2-dibromoethane, 1,3-dibromopropane, 1,4-dibromobutane, 1,6-dibromohexane or 1,8-dibromooctane in 12 mL of methanol. Then 60 mmol of 1-methylimidazole was added dropwise while stirring at room temperature. The resulting mixture was further heated and stirred at 40–50 °C for 48 hours. The product was isolated by filtration and purified by recrystallization. The resulting product was transferred to a single-necked round-bottomed flask, washing with methanol. The solvent was then removed under reduced pressure using a rotary evaporator. Data in agreement with the literature [15], [16], [17], [18].

**C**_**3**_ **(MPyrr)**_**2**_ **Br**_**2**_, **C**_**4**_ **(MPyrr)**_**2**_ **Br**_**2**_, **C**_**6**_ **(MPyrr)**_**2**_ **Br**_**2**_ and **C**_**8**_ **(MPyrr)**_**2**_ **Br**_**2**_ were synthesized with the same procedure [15], [19]. A three-necked round-bottomed flask fitted with a reflux condenser and pressure-equalised funnel was charged with a solution of 30 mmol of 1,3-dibromopropane, 1,4-dibromobutane, 1,6-dibromohexane or 1,8-dibromooctane in 10 ml of methanol. Then 63 mmol of 1-methylimidazole was added dropwise while stirring at room temperature. The resulting mixture was further heated and stirred at 40–50 °C for 48 hours. The product was isolated by filtration and purified by recrystallization in methanol/ethyl acetate (≈1:3 v:v). The resulting product was transferred to a single-necked round-bottomed flask, washing with methanol. The solvent was then removed under reduced pressure using a rotary evaporator. Data in agreement with the literature [15], [18].

**C**_**2**_ **(Pyr)**_**2**_ **Br**_**2**_, **C**_**3**_ **(Pyr)**_**2**_ **Br**_**2**_, **C**_**4**_ **(Pyr)**_**2**_ **Br**_**2**_, **C**_**6**_ **(Pyr)**_**2**_ **Br**_**2**_, **C**_**8**_ **(Pyr)**_**2**_ **Br**_**2**_ and **C**_**12**_ **(Pyr)**_**2**_ **Br**_**2**_ were synthesized with the same procedure [15]. A three-necked round bottomed flask fitted with a reflux condenser and pressure-equalised addition funnel was charged with a solution of 30 mmol of 1,2-dibromoethane, 1,3-dibromopropane, 1,4-dibromobutane, 1,6-dibromohexane, 1,8-dibromooctane or 1,12-dibromododecane in 5 mL of methanol. Then 75 mmol of 1-methylimidazole was added dropwise while stirring at room temperature. The resulting mixture was further heated and stirred at 50 °C for 48 hours. The product was isolated by filtration and purified by recrystallization in methanol/ethyl acetate (≈1:3 v:v). The resulting product was transferred to a single-necked round-bottomed flask washing with methanol. The solvent was then removed under reduced pressure using a rotary evaporator. Data in agreement with the literature [15].

**1-(3-bromopropyl)pyridinium bromide** was synthesized following the procedure described in the literature [20]. 100 mmol of pyridine was transferred to a round bottom flask. 150 mmol of 1,3-dibromopropane was added and the resulting mixture was stirred for three days at room temperature. Then, the mixture was washed with ethyl acetate to remove any unreacted reactants and filtered to obtain a white precipitate.

**C**_**3**_ **(Pyr) (MIm) Br**_**2**_ and **C**_**3**_ **(Pyr) (MPyrr) Br**_**2**_ were synthesized with the same procedure [20]. A three-neck round bottom flask fitted with a reflux condenser was charged with a solution of 15 mmol of (3-bromopropyl)pyridinium bromide in 20 mL of methanol. Then 18 mmol of 1-methylimidazole or 19.5 mmol of 1-methylpyrrolidine was added dropwise while stirring at room temperature. The resulting mixture was refluxed while stirring at 50 °C for 48 hours. The resulting solution was recrystallised directly from methanol/ethyl acetate (≈1:5 v:v) and the product was isolated by filtration.

The structures of the resulting ILs were confirmed by ^1^H, ^13^C and ^19^F NMR spectroscopy (recorded generally at room temperature on a Jeol model EX270) and mass spectrometry (Bruker MicroTOF 61 spectrometer).

**C**_**8**_**(MIm)Br:**1-methyl-3-octylimidazolium bromide. C_12_H_23_BrN_2_; from 8.21 g (100 mmol) of 1-methylimidazole and 21.24 g (110 mmol) of 1-bromooctane, 22.98 g (83.49 mmol) of C_8_(MIm)Br was obtained (yield: 84%); ^1^H NMR (270 MHz, CDCl_3_): δ 10.29 (s, 1H, N-C*H*-N), 7.62 (s, 1H, N-C*H*-CH-N), 7.43 (s, 1H, N-CH-C*H*-N), 4.27 (t, 2H, N-C*H*_*2*_-CH_2_), 4.08 (s, 3H, N-C*H*_*3*_), 1.91-1.80 (m, 2H, N-CH_2_-C*H*_*2*_-C_5_H_10_-CH_3_), 1.31-1.15 (m, 10H, N-CH_2_-CH_2_-C_5_*H*_*10*_-CH_3_), 0.80 (t, 3H, N-CH_2_-CH_2_-C_5_H_10_-C*H*_*3*_). ^13^C NMR (68 MHz, CDCl_3_): δ 137.49 (N-*C*H-N), 123.97 (N-*C*H-CH-N), 122.21 (N-CH-*C*H-N), 50.31 (N-*C*H_2_-CH_2_), 36.91 (N-*C*H_3_), 31.83 (N-C_5_H_10_-*C*H_2_-CH_2_-CH_3_), 30.50 (N-CH_2_-*C*H_2_-C_5_H_10_-CH_3_), 29.18 (N-C_4_H_8_-*C*H_2_-C_2_H_4_-CH_3_), 29.11 (N-C_3_H_6_-*C*H_2_-C_3_H_6_-CH_3_), 26.41 (N-C_2_H_4_-*C*H_2_-C_4_H_8_-CH_3_), 22.73 (N-C_6_H_12_-*C*H_2_-CH_3_), 14.24 (N-C_7_H_14_-*C*H_3_). MS m/z molecular ion: calcd. 195.1856; found 195.1864 (cation).

**C**_**8**_**(MPyrr)Br:** 1-methyl-1-octylpyrrolidinium bromide. C_13_H_28_BrN; from 20.44 g (240 mmol) of 1-methylpyrrolidine and 55.62 g (288 mmol) of 1-bromooctane, 62.61 g (224.99 mmol) of C_8_(MPyrr)Br was obtained (yield: 94%); ^1^H NMR (270 MHz, CDCl_3_): δ 3.83-3.74 (m, 4H, N-C*H*_*2*_-CH_2_-CH_2_-C*H*_*2*_-N), 3.62-3.56 (m, 2H, N-C*H*_*2*_-CH_2_-C_5_H_10_-CH_3_), 3.24 (s, 3H, N-C*H*_*3*_), 2.28-2.20 (m, 4H, N-CH_2_-C*H*_*2*_-C*H*_*2*_-CH_2_-N), 1.77-1.66 (m, 2H, N-CH_2_-C*H*_*2*_-C_5_H_10_-CH_3_), 1.36-1.14 (m, 10H, N-CH_2_-CH_2_-C_5_*H*_*10*_-CH_3_), 0.81 (t, 3H, N-CH_2_-CH_2_-C_5_H_10_-C*H*_*3*_). ^13^C NMR (68 MHz, CDCl_3_): δ 64.66 (N-*C*H_2_-CH_2_-CH_2_-*C*H_2_-N), 64.41 (N-*C*H_2_-C_6_H_12_-CH_3_), 48.89 (N-*C*H_3_), 31.82 (N-C_5_H_10_-*C*H_2_-CH_2_-CH_3_), 29.37 (N-C_4_H_8_-*C*H_2_-C_2_H_4_-CH_3_), 29.20 (N-C_3_H_6_-*C*H_2_-C_3_H_6_-CH_3_), 26.61 (N-C_2_H_4_-*C*H_2_-C_4_H_8_-CH_3_), 24.31 (N-CH_2_-*C*H_2_-C_5_H_10_-CH_3_), 22.74 (N-C_6_H_12_-*C*H_2_-CH_3_), 21.87 (N-CH_2_-C*H*_*2*_-C*H*_*2*_-CH_2_-N), 14.24 (N-C_7_H_14_-*C*H_3_). MS m/z molecular ion: calcd. 198.2216; found 198.2227 (cation).

**C**_**8**_**(Pyr)Br:** 1-octylpyridinium bromide. C_13_H_22_BrN; from 18.98 g (240 mmol) of pyridine and 55.62 g (288 mmol) of 1-bromooctane, 57.53 g (211.34 mmol) of C_8_(Pyr)Br was obtained (yield: 88%); ^1^H NMR (270 MHz, CDCl_3_): δ 9.54 (d, 2H, N-C*H*-CH-CH-CH-C*H*-N), 8.50 (t, 1H, N-CH-CH-C*H*-CH-CH-N), 8.13 (t, 2H, N-CH-C*H*-CH-C*H*-CH-N), 4.93 (t, 2H, N-C*H*_*2*_-CH_2_-C_5_H_10_-CH_3_), 2.02-1.95 (m, 2H, N-CH_2_-C*H*_*2*_-C_5_H_10_-CH_3_), 1.33-1.09 (m, 10H, N-CH_2_-CH_2_-C_5_*H*_*10*_-CH_3_), 0.77 (t, 3H, N-CH_2_-CH_2_-C_5_H_10_-C*H*_*3*_). ^13^C NMR (68 MHz, CDCl_3_): δ 145.59 (N-CH-CH-C*H*-CH-CH-N), 145.26 (N-C*H*-CH-CH-CH-C*H*-N), 128.82 (N-CH-C*H*-CH-C*H*-CH-N), 62.36 (N-*C*H_2_-C_6_H_12_-CH_3_), 32.15 (N-C_5_H_10_-*C*H_2_-CH_2_-CH_3_), 31.89 (N-CH_2_-*C*H_2_-C_5_H_10_-CH_3_), 29.22 (N-C_3_H_6_-*C*H_2_-*C*H_2_-C_2_H_4_-CH_3_), 26.28 (N-C_2_H_4_-*C*H_2_-C_4_H_8_-CH_3_), 22.78 (N-C_6_H_12_-*C*H_2_-CH_3_), 14.18 (N-C_7_H_14_-*C*H_3_). MS m/z molecular ion: calcd. 192.1747; found 192.1753 (cation).

**C**_**8**_**(MIm)NTf**_**2**_**:**1-methyl-3-octylimidazolium bis (trifluoromethylsulfonyl) imide. C_14_H_23_F_6_N_3_O_4_S_2_; from 4.04 g (14.68 mmol) of C_8_(MIm)Br and 5.06 g (17.62 mmol) of LiNTf_2_, 6.91 g (14.53 mmol) of C_8_(MIm)NTf_2_ was obtained (yield: 99%);^1^H NMR (270 MHz, CD_4_O): δ 8.87 (s, 1H, N-C*H*-N), 7.58 (d, 2H, N-C*H*-C*H*-N), 4.20 (t, 2H, N-C*H*_*2*_-CH_2_), 3.92 (s, 3H, N-C*H*_*3*_), 1.94-1.83 (m, 2H, N-CH_2_-C*H*_*2*_-C_5_H_10_-CH_3_), 1.37-1.28 (m, 10H, N-CH_2_-CH_2_-C_5_*H*_*10*_-CH_3_), 0.90 (t, 3H, N-CH_2_-CH_2_-C_5_H_10_-C*H*_*3*_). ^13^C NMR (68 MHz, CD_4_O): δ 137.33 (N-*C*H-N), 125.05 (N-*C*H-CH-N), 123.75 (N-CH-*C*H-N), 50.98 (N-*C*H_2_-CH_2_), 36.54 (N-*C*H_3_), 33.01 (N-CH_2_-*C*H_2_-C_5_H_10_-CH_3_), 31.23 (N-C_5_H_10_-*C*H_2_-CH_2_-CH_3_), 30.30 (N-C_4_H_8_-*C*H_2_-C_2_H_4_-CH_3_), 30.16 (N-C_3_H_6_-*C*H_2_-C_3_H_6_-CH_3_), 27.38 (N-C_2_H_4_-*C*H_2_-C_4_H_8_-CH_3_), 23.79 (N-C_6_H_12_-*C*H_2_-CH_3_), 14.52 (N-C_7_H_14_-*C*H_3_). ^19^F NMR (200 MHz, CD_4_O) δ −81.1 ppm. MS m/z molecular ion: calcd. 195.1856; found 195.1863 (cation), calcd. 279.9178; found 279.9180 (anion).

**C**_**8**_**(MPyrr)NTf**_**2**_**:**1-methyl-1-octylpyrrolidinium bis (trifluoromethylsulfonyl) imide. C_15_H_28_F_6_N_2_O_4_S_2_; from 1.40 g (5.03 mmol) of C_8_(MPyrr)Br and 1.73 g (6.04 mmol) of LiNTf_2_, 2.34 g (4.89 mmol) of C_8_(MPyrr)NTf_2_ was obtained (yield: 97%); ^1^H NMR (270 MHz, CDCl_3_): δ 3.57-3.45 (m, 4H, N-C*H*_*2*_-CH_2_-CH_2_-C*H*_*2*_-N), 3.32-3.26 (m, 2H, N-C*H*_*2*_-CH_2_-C_5_H_10_-CH_3_), 3.03 (s, 3H, N-C*H*_*3*_), 2.31-2.20 (m, 4H, N-CH_2_-C*H*_*2*_-C*H*_*2*_-CH_2_-N), 1.80-1.71 (m, 2H, N-CH_2_-C*H*_*2*_-C_5_H_10_-CH_3_), 1.38-1.21 (m, 10H, N-CH_2_-CH_2_-C_5_*H*_*10*_-CH_3_), 0.88 (t, 3H, N-CH_2_-CH_2_-C_5_H_10_-C*H*_*3*_). ^13^C NMR (68 MHz, CDCl_3_): δ 65.14 (N-*C*H_2_-CH_2_-CH_2_-*C*H_2_-N), 64.87 (N-*C*H_2_-C_6_H_12_-CH_3_), 48.69 (N-*C*H_3_), 31.87 (N-C_5_H_10_-*C*H_2_-CH_2_-CH_3_), 29.22 (N-C_3_H_6_-*C*H_2_-*C*H_2_-C_2_H_4_-CH_3_), 24.14 (N-CH_2_-*C*H_2_-C_5_H_10_-CH_3_), 22.85 (N-C_6_H_12_-*C*H_2_-CH_3_), 21.81 (N-CH_2_-C*H*_*2*_-C*H*_*2*_-CH_2_-N), 14.32 (N-C_7_H_14_-*C*H_3_). ^19^F NMR (200 MHz, CDCl_3_) δ −79.5 ppm. MS m/z molecular ion: calcd. 198.2216; found 198.2227 (cation), calcd. 279.9178; found 279.9179 (anion).

**C**_**8**_**(Pyr)NTf**_**2**_**:** 1-octylpyridinium bis (trifluoromethylsulfonyl) imide. C_15_H_22_F_6_N_2_O_4_S_2_; from 1.36 g (5 mmol) of C_8_(Pyr)Br and 1.72 g (6 mmol) of LiNTf_2_, 2.21 g (4.68 mmol) of C_8_(Pyr)NTf_2_ was obtained (yield: 94%); ^1^H NMR (270 MHz, CDCl_3_): δ 8.83 (d, 2H, N-C*H*-CH-CH-CH-C*H*-N), 8.48 (t, 1H, N-CH-CH-C*H*-CH-CH-N), 8.06 (t, 2H, N-CH-C*H*-CH-C*H*-CH-N), 4.59 (t, 2H, N-C*H*_*2*_-CH_2_-C_5_H_10_-CH_3_), 2.05-1.94 (m, 2H, N-CH_2_-C*H*_*2*_-C_5_H_10_-CH_3_), 1.40-1.19 (m, 10H, N-CH_2_-CH_2_-C_5_*H*_*10*_-CH_3_), 0.86 (t, 3H, N-CH_2_-CH_2_-C_5_H_10_-C*H*_*3*_). ^13^C NMR (68 MHz, CDCl_3_): δ 145.75 (N-CH-CH-C*H*-CH-CH-N), 144.71 (N-C*H*-CH-CH-CH-C*H*-N), 128.99 (N-CH-C*H*-CH-C*H*-CH-N), 62.97 (N-*C*H_2_-C_6_H_12_-CH_3_), 31.87 (N-CH_2_-*C*H_2_-C_3_H_6_-*C*H_2_-CH_2_-CH_3_), 29.17 (N-C_4_H_8_-*C*H_2_-C_2_H_4_-CH_3_), 29.08 (N-C_3_H_6_-*C*H_2_-C_3_H_6_-CH_3_), 26.23 (N-C_2_H_4_-*C*H_2_-C_4_H_8_-CH_3_), 22.83 (N-C_6_H_12_-*C*H_2_-CH_3_), 14.30 (N-C_7_H_14_-*C*H_3_). ^19^F NMR (200 MHz, CDCl_3_) δ −79.5 ppm. MS m/z molecular ion: calcd. 192.1747; found 192.1742 (cation), calcd. 279.9178; found 279.9188 (anion).

**C**_**8**_**(MIm)SbF**_**6**_**:**1-methyl-3-octylimidazolium hexafluoroantimonate. C_12_H_23_F_6_N_2_Sb; from 1.35 g (4.92 mmol) of C_8_(MIm)Br and 1.56 g (6.01 mmol) of NaSbF_6_, 1.93 g (4.48 mmol) of C_8_(MIm)SbF_6_ was obtained (yield: 91%); ^1^H NMR (270 MHz, DMSO-d6): δ 9.09 (s, 1H, N-C*H*-N), 7.72 (d, 2H, N-C*H*-C*H*-N), 4.14 (t, 2H, N-C*H*_*2*_-CH_2_), 3.84 (s, 3H, N-C*H*_*3*_), 1.82-1.73 (m, 2H, N-CH_2_-C*H*_*2*_-C_5_H_10_-CH_3_), 1.32-1.18 (m, 10H, N-CH_2_-CH_2_-C_5_*H*_*10*_-CH_3_), 0.86 (t, 3H, N-CH_2_-CH_2_-C_5_H_10_-C*H*_*3*_). ^13^C NMR (68 MHz, DMSO-d6): δ 136.47 (N-*C*H-N), 123.58 (N-*C*H-CH-N), 122.24 (N-CH-*C*H-N), 48.77 (N-*C*H_2_-CH_2_), 35.72 (N-*C*H_3_), 31.15 (N-CH_2_-*C*H_2_-C_5_H_10_-CH_3_), 29.35 (N-C_5_H_10_-*C*H_2_-CH_2_-CH_3_), 28.45 (N-C_4_H_8_-*C*H_2_-C_2_H_4_-CH_3_), 28.32 (N-C_3_H_6_-*C*H_2_-C_3_H_6_-CH_3_), 25.47 (N-C_2_H_4_-*C*H_2_-C_4_H_8_-CH_3_), 22.04 (N-C_6_H_12_-*C*H_2_-CH_3_), 13.91 N-C_7_H_14_-*C*H_3_). ^19^F NMR (200 MHz, DMSO-d6, at −40 °C) δ −121.4 ppm. MS m/z molecular ion: calcd. 195.1856; found 195.1865 (cation), calcd. 234.8948; found 234.8935 (anion).

**C**_**8**_**(MPyrr)SbF**_**6**_**:** 1-methyl-1-octylpyrrolidinium hexafluoroantimonate. C_13_H_28_F_6_NSb; from 1.40 g (5.03 mmol) of C_8_(MPyrr)Br and 1.56 g (6.03 mmol) of NaSbF_6_, 2.05 g (4.72 mmol) of C_8_(MPyrr)SbF_6_ was obtained (yield: 94%); ^1^H NMR (270 MHz, DMSO-d6): δ 3.51-3.34 (m, 4H, N-C*H*_*2*_-CH_2_-CH_2_-C*H*_*2*_-N), 3.31-3.23 (m, 2H, N-C*H*_*2*_-CH_2_-C_5_H_10_-CH_3_), 2.97 (s, 3H, N-C*H*_*3*_), 2.13-2.02 (m, 4H, N-CH_2_-C*H*_*2*_-C*H*_*2*_-CH_2_-N), 1.74-1.63 (m, 2 H, N-CH_2_-C*H*_*2*_-C_5_H_10_-CH_3_), 1.35-1.23 (m, 10H, N-CH_2_-CH_2_-C_5_*H*_*10*_-CH_3_), 0.87 (t, 3H, N-CH_2_-CH_2_-C_5_H_10_-C*H*_*3*_). ^13^C NMR (68 MHz, DMSO-d6): δ 63.38 (N-*C*H_2_-CH_2_-CH_2_-*C*H_2_-N), 63.09 (N-*C*H_2_-C_6_H_12_-CH_3_), 47.46 (N-*C*H_3_), 31.14 (N-C_5_H_10_-*C*H_2_-CH_2_-CH_3_), 28.43 (N-C_3_H_6_-*C*H_2_-*C*H_2_-C_2_H_4_-CH_3_), 25.89 (N-CH_2_-*C*H_2_-C_5_H_10_-CH_3_), 22.88 (N-C_6_H_12_-*C*H_2_-CH_3_), 22.01 N-CH_2_-C*H*_*2*_-CH_2_-CH_2_-N), 21.05 (N-CH_2_-CH_2_-C*H*_*2*_-CH_2_-N), 13.91 (N-C_7_H_14_-*C*H_3_). ^19^F NMR (200 MHz, DMSO-d6, at −40 °C) δ -120.5 ppm. MS m/z molecular ion: calcd. 198.2216; found 198.2225 (cation), calcd. 234.8948; found 234.8939 (anion).

**C**_**8**_**(Pyr)SbF**_**6**_**:**1-octylpyridinium hexafluoroantimonate. C_13_H_22_F_6_NSb; from 1.37 g (5 mmol) of C_8_(Pyr)Br and 1.56 g (6 mmol) of NaSbF_6_, 2.00 g (4.67 mmol) of C_8_(Pyr)SbF_6_ was obtained (yield: 93%); ^1^H NMR (270 MHz, DMSO-d6): δ 9.08 (d, 2H, N-C*H*-CH-CH-CH-C*H*-N), 8.60 (t, 1H, N-CH-CH-C*H*-CH-CH-N), 8.16 (t, 2H, N-CH-C*H*-CH-C*H*-CH-N), 4.59 (t, 2 H, N-C*H*_*2*_-CH_2_-C_5_H_10_-CH_3_), 1.97-1.86 (m, 2H, N-CH_2_-C*H*_*2*_-C_5_H_10_-CH_3_), 1.33-1.20 (m, 10H, N-CH_2_-CH_2_-C_5_*H*_*10*_-CH_3_), 0.85 (t, 3H, N-CH_2_-CH_2_-C_5_H_10_-C*H*_*3*_). ^13^C NMR (68 MHz, DMSO-d6): δ 145.45 (N-CH-CH-C*H*-CH-CH-N), 144.72 N-C*H*-CH-CH-CH-C*H*-N), 128.08 (N-CH-C*H*-CH-C*H*-CH-N), 60.80 (N-*C*H_2_-C_6_H_12_-CH_3_), 31.11 (N-C_5_H_10_-*C*H_2_-CH_2_-CH_3_), 30.69 (N-CH_2_-*C*H_2_-C_5_H_10_-CH_3_), 28.42 (N-C_4_H_8_-*C*H_2_-C_2_H_4_-CH_3_), 28.33 (N-C_3_H_6_-*C*H_2_-C_3_H_6_-CH_3_), 25.39 (N-C_2_H_4_-*C*H_2_-C_4_H_8_-CH_3_), 22.03 (N-C_6_H_12_-*C*H_2_-CH_3_), 13.92 (N-C_7_H_14_-*C*H_3_). ^19^F NMR (200 MHz, DMSO-d6, at −40 °C) δ −118.7 ppm. MS m/z molecular ion: calcd. 192.1747; found 192.1737 (cation), calcd. 234.8948; found 234.8943(anion).

**C**_**2**_ **(MIm)**_**2**_ **Br**_**2**_**:** 1,2-bis(3-methylimidazolium-1-yl)ethane dibromide. C_10_H_16_Br_2_N_4_; from 4.93 g (60 mmol) of 1-methylimidazole and 5.64 g (30 mmol) of 1,2-dibromoethane, 3.74 g (10.62 mmol) of C_2_ (MIm)_2_ Br_2_ was obtained (yield: 35%); ^1^H NMR (270 MHz, DMSO-d6): δ 9.25 (s, 2H, N-C*H*-N), 7.75 (d, 4H, N-C*H*-C*H*-N), 4.76 (s, 4H, N-C*H*_*2*_-C*H*_*2*_-N), 3.86 (s, 6H, N-C*H*_*3*_). ^13^C NMR (68 MHz, DMSO-d6): δ 137.16 (N-*C*H-N), 123.78 (N-*C*H-CH-N), 122.33 (N-CH-*C*H-N), 48.30 (N-*C*H_2_-*C*H_2_-N), 36.01 (N-*C*H_3_). MS m/z molecular ion: calcd. 192.1371; found 192.1347 (cation^2+^).

**C**_**3**_ **(MIm)**_**2**_ **Br**_**2**_**:** 1,3-bis(3-methylimidazolium-1-yl)propane dibromide. C_11_H_18_Br_2_N_4_; from 4.93 g (60 mmol) of 1-methylimidazole and 6.06 g (30 mmol) of 1,3-dibromopropane, 10.31 (28.16 mmol) g of C_3_ (MIm)_2_ Br_2_ was obtained (yield: 94%); ^1^H NMR (270 MHz, D_2_O): δ 8.78 (s, 2H, N-C*H*-N), 7.47 (d, 4H, N-C*H*-C*H*-N), 4.30 (t, 4H, N-C*H*_*2*_-CH_2_-C*H*_*2*_-N), 3.88 (s, 6H, N-C*H*_*3*_), 2.56-2.45 (m, 2H, N-CH_2_-C*H*_*2*_-CH_2_-N). ^13^C NMR (68 MHz, D_2_O): δ 136.38 (N-*C*H-N), 124.16 (N-*C*H-CH-N), 122.36 (N-CH-*C*H-N), 46.49 (N-*C*H_2_-CH_2_-*C*H_2_-N), 36.10 (N-*C*H_3_), 29.95 (N-CH_2_-*C*H_2_-CH_2_-N). MS m/z molecular ion: calcd. 103.0730; found 103.0727 (cation^+^).

**C**_**4**_ **(MIm)**_**2**_ **Br**_**2**_**:** 1,4-bis(3-methylimidazolium-1-yl)butane dibromide. C_12_H_20_Br_2_N_4_; from 4.93 g (60 mmol) of 1-methylimidazole and 6.48 g (30 mmol) of 1,4-dibromobutane, 7.85 g (20.65 mmol) of C_4_ (MIm)_2_ Br_2_ was obtained (yield: 69%); ^1^H NMR (270 MHz, D_2_O): δ 8.70 (s, 2H, N-C*H*-N), 7.42 (d, 4H, N-C*H*-C*H*-N), 4.21 (t, 4H, N-C*H*_*2*_-C_2_H_4_-C*H*_*2*_-N), 3.85 (s, 6H, N-C*H*_*3*_), 1.90-1.84 (m, 4H, N-CH_2_-C_2_*H*_*4*_-CH_2_-N). ^13^C NMR (68 MHz, D_2_O): δ 136.16 (N-*C*H-N), 123.92 (N-*C*H-CH-N), 122.28 (N-CH-*C*H-N), 48.92 (N-*C*H_2_-C_2_H_4_-*C*H_2_-N), 35.97 (N-*C*H_3_), 26.41 (N-CH_2_-*C*_*2*_H_4_-CH_2_-N). MS m/z molecular ion: calcd. 110.0812; found 110.0805 (cation^+^).

**C**_**6**_ **(MIm)**_**2**_ **Br**_**2**_**:** 1,6-bis(3-methylimidazolium-1-yl)hexane dibromide. C_14_H_24_Br_2_N_4_; from 4.93 g (60 mmol) of 1-methylimidazole and 7.32 g (30 mmol) of 1,6-dibromohexane, 11.26 g (27.59 mmol) of C_6_ (MIm)_2_ Br_2_ was obtained (yield: 92%); ^1^H NMR (270 MHz, DMSO-d6): δ 9.31 (s, 2H, N-C*H*-N), 7.80 (d, 4H, N-C*H*-C*H*-N), 4.19 (t, 4H, N-C*H*_*2*_-C_4_H_8_-C*H*_*2*_-N), 3.87 (s, 6H, N-C*H*_*3*_), 1.81-1.76 (m, 4H, N-CH_2_-C*H*_*2*_-C_2_H_4_-C*H*_*2*_-CH_2_-N), 1.29-1.24 (m, 4H, N-C_2_H_4_-C_2_*H*_*4*_-C_2_H_4_-N). ^13^C NMR (68 MHz, DMSO-d6): δ 136.50 (N-*C*H-N), 123.53 (N-*C*H-CH-N), 122.24 (N-CH-*C*H-N), 48.53 (N-*C*H_2_-C_4_H_8_-*C*H_2_-N), 35.77 (N-*C*H_3_), 29.07 (N-CH_2_-*C*H_2_-C_2_H_4_-*C*H_2_-CH_2_-N), 24.76 (N-C_2_H_4_-*C*_*2*_H_4_-C_2_H_4_-N). MS m/z molecular ion: calcd. 124.0997; found 124.0985 (cation^+^).

**C**_**8**_ **(MIm)**_**2**_ **Br**_**2**_**:** 1,8-bis(3-methylimidazolium-1-yl)octane dibromide. C_16_H_28_Br_2_N_4_; from 4.93 g (60 mmol) of 1-methylimidazole and 8.16 g (30 mmol) of 1,8-dibromooctane, 12.90 g (29.57 mmol) of C_8_ (MIm)_2_ Br_2_ was obtained (yield: 99%); ^1^H NMR (270 MHz, D_2_O): δ 8.66 (s, 2H, N-C*H*-N), 7.40 (d, 4H, N-C*H*-C*H*-N), 4.14 (t, 4H, N-C*H*_*2*_-C_4_H_8_-C*H*_*2*_-N), 3.84 (s, 6H, N-C*H*_*3*_), 1.86-1.76 (m, 4H, N-CH_2_-C*H*_*2*_-C_4_H_8_-C*H*_*2*_-CH_2_-N), 1.30-1.18 (m, 8H, N-C_2_H_4_-C_4_*H*_*8*_-C_2_H_4_-N). ^13^C NMR (68 MHz, D_2_O): δ 135.97 (N-*C*H-N), 123.65 (N-*C*H-CH-N), 122.67 (N-CH-*C*H-N), 49.70 (N-*C*H_2_-C_6_H_12_-*C*H_2_-N), 35.85 (N-*C*H_3_), 29.33 (N-CH_2_-*C*H_2_-C_4_H_8_-*C*H_2_-CH_2_-N), 28.02 (N-C_2_H_4_-*C*H_2_-C_4_H_8_-*C*H_2_-C_2_H_4_-N), 25.38 (N-C_3_H_6_-*C*_*2*_H_4_-C_3_H_6_-N). MS m/z molecular ion: calcd. 138.1151; found 138.1155 (cation^+^).

**C**_**3**_ **(MPyrr)**_**2**_ **Br**_**2**_**:** 1,3-bis(1-methylpyrrolidinium-1-yl)propane dibromide. C_13_H_28_Br_2_N_2_; from 5.36 g (63 mmol) of 1-methylpyrrolidine and 6.06 g (30 mmol) of 1,3-dibromopropane, 10.28 g (27.62 mmol) of C_3_ (MPyrr)_2_ Br_2_ was obtained (yield: 92%); ^1^H NMR (270 MHz, D_2_O): δ 3.65-3.41 (m, 12H, N-C*H*_*2*_-CH_2_), 3.10 (s, 6H, N-C*H*_*3*_), 2.44-2.31 (m, 2H, N-CH_2_-C*H*_*2*_-CH_2_-N), 2.28-2.17 (m, 8H, N-CH_2_-C*H*_*2*_-C*H*_*2*_-CH_2_-N). ^13^C NMR (68 MHz, D_2_O): δ 65.09 (N-*C*H_2_-CH_2_-CH_2_-*C*H_2_-N), 60.46 (N-*C*H_2_-CH_2_-*C*H_2_-N), 48.51 (N-*C*H_3_), 21.52 (N-CH_2_-*C*H_2_-*C*H_2_-CH_2_-N), 19.13 (N-CH_2_-*C*H_2_-CH_2_-N). MS m/z molecular ion: calcd. 106.1123; found 106.1111 (cation^+^).

**C**_**4**_ **(MPyrr)**_**2**_ **Br**_**2**_**:** 1,4-bis(1-methylpyrrolidinium-1-yl)butane dibromide. C_14_H_30_Br_2_N_2_; from 5.36 g (63 mmol) of 1-methylpyrrolidine and 6.48 g (30 mmol) of 1,4-dibromobutane, 8.04 g (20.82 mmol) of C_4_ (MPyrr)_2_ Br_2_ was obtained (yield: 69%); ^1^H NMR (270 MHz, D_2_O): δ 3.54-3.37 (m, 12H, N-C*H*_*2*_-CH_2_), 3.02 (s, 6 H, N-C*H*_*3*_), 2.21-2.14 (m, 8H, N-CH_2_-C*H*_*2*_-C*H*_*2*_-CH_2_-N ring), 1.90-1.84 (m, 4 H, N-CH_2_-C*H*_*2*_-C*H*_*2*_-CH_2_-N alkyl linkage chain). ^13^C NMR (68 MHz, D_2_O): δ 64.67 (N-*C*H_2_-CH_2_-CH_2_-*C*H_2_-N ring), 63.32 (N-*C*H_2_-CH_2_-CH_2_-*C*H_2_-N alkyl linkage chain), 48.29 (N-*C*H_3_), 21.47 (N-CH_2_-*C*H_2_-*C*H_2_-CH_2_-N ring), 20.66 (N-CH_2_-*C*H_2_-*C*H_2_-CH_2_-N alkyl linkage chain). MS m/z molecular ion: calcd. 113.1201; found 113.1187 (cation^+^).

**C**_**6**_ **(MPyrr)**_**2**_ **Br**_**2**_**:** 1,6-bis(1-methylpyrrolidinium-1-yl)hexane dibromide. C_16_H_34_Br_2_N_2_; from 5.36 g (63 mmol) of 1-methylpyrrolidine and 7.32 g (30 mmol) of 1,6-dibromohexane, 11.68 g (29.18 mmol) of C_6_ (MPyrr)_2_ Br_2_ was obtained (yield: 94%); ^1^H NMR (270 MHz, D_2_O): δ 3.51-3.27 (m, 12H, N-C*H*_*2*_-CH_2_), 3.00 (s, 6H, N-C*H*_*3*_), 2.22-2.11 (m, 8H, N-CH_2_-C*H*_*2*_-C*H*_*2*_-CH_2_-N), 1.85-1.73 (m, 4H, N-CH_2_-C*H*_*2*_-C_2_H_4_-C*H*_*2*_-CH_2_-N), 1.43-1.37 (m, 4H, N-C_2_H_4_-C_2_*H*_*4*_-C_2_H_4_-N). ^13^C NMR (68 MHz, D_2_O): δ 64.48 (N-*C*H_2_-CH_2_-CH_2_-*C*H_2_-N), 64.22 (N-*C*H_2_-C_4_H_6_-*C*H_2_-N), 48.27 (N-*C*H_3_), 25.47 (N-C_2_H_4_-*C*_*2*_H_4_-C_2_H_4_-N), 23.18 (N-CH_2_-*C*H_2_-C_2_H_4_-*C*H_2_-CH_2_-N), 21.48 (N-CH_2_-*C*H_2_-*C*H_2_-CH_2_-N). MS m/z molecular ion: calcd. 127.1357; found 127.1315 (cation^+^).

**C**_**8**_ **(MPyrr)**_**2**_ **Br**_**2**_**:** 1,8-bis(1-methylpyrrolidinium-1-yl)octane dibromide. C_18_H_38_Br_2_N_2_; from 5.36 g (63 mmol) of 1-methylpyrrolidine and 8.16 g (30 mmol) of 1,8-dibromooctane, 12.63 g (28.55 mmol) of C_8_ (MPyrr)_2_ Br_2_ was obtained (yield: 95%); ^1^H NMR (270 MHz, D_2_O): δ 3.53-3.25 (m, 12H, N-C*H*_*2*_-CH_2_), 2.99 (s, 6H, N-C*H*_*3*_), 2.23-2.10 (m, 8H, N-CH_2_-C*H*_*2*_-C*H*_*2*_-CH_2_-N), 1.81-1.70 (m, 4H, N-CH_2_-C*H*_*2*_-C_4_H_8_-C*H*_*2*_-CH_2_-N), 1.34 (s, 8H, N-C_2_H_4_-C_4_*H*_*8*_-C_2_H_4_-N). ^13^C NMR (68 MHz, D_2_O): δ 64.46 (N-*C*H_2_-CH_2_-CH_2_-*C*H_2_-N), 64.42 (N-*C*H_2_-C_6_H_12_-*C*H_2_-N), 48.25 (N-*C*H_3_), 28.10 (N-C_3_H_6_-*C*_*2*_H_4_-C_3_H_6_-N), 25.70 (N-C_2_H_4_-*C*H_2_-C_2_H_4_-*C*H_2_-C_2_H_4_-N), 23.31 (N-CH_2_-*C*H_2_-C_4_H_8_-*C*H_2_-CH_2_-N), 21.47 (N-CH_2_-*C*H_2_-*C*H_2_-CH_2_-N). MS m/z molecular ion: calcd. 141.1488; found 141.1491 (cation^+^).

**C**_**2**_ **(Pyr)**_**2**_ **Br**_**2**_**:** 1,2-bis(pyridinium-1-yl)ethane dibromide. C_12_H_14_Br_2_N_2_; from 5.93 g (75 mmol) of pyridine and 5.64 g (30 mmol) of 1,2-dibromoethane, 5.96 g (17.22 mmol) of C_2_ (Pyr)_2_ Br_2_ was obtained (yield: 57%); ^1^H NMR (270 MHz, D_2_O): δ 8.84 (d, 4H, N-C*H*-CH), 8.65 (t, 2H, N-CH-CH-C*H*-CH-CH-N), 8.11 (t, 4H, N-CH-C*H*-CH-C*H*-CH-N), 5.34 (s, 4H, N-C*H*_*2*_-C*H*_*2*_-N). ^13^C NMR (68 MHz, D_2_O): δ 147.74 (N-CH-CH-*C*H-CH-CH-N), 144.93 (N-*C*H-CH-CH-CH-*C*H-N), 129.39 (N-CH-*C*H-CH-*C*H-CH-N), 60.31 (N-*C*H_2_-*C*H_2_-N). MS m/z molecular ion: calcd. 186.1153; found 186.1165 (cation^2+^).

**C**_**3**_ **(Pyr)**_**2**_ **Br**_**2**_**:** 1,3-bis(pyridinium-1-yl)propane dibromide. C_13_H_16_Br_2_N_2_; from 5.93 g (75 mmol) of pyridine and 6.06 g (30 mmol) of 1,3-dibromopropane, 10.42 g (28.94 mmol) of C_3_ (Pyr)_2_ Br_2_ was obtained (yield: 96%); ^1^H NMR (270 MHz, D_2_O): δ 8.90 (d, 4H, N-C*H*-CH), 8.57 (t, 2H, N-CH-CH-C*H*-CH-CH-N), 8.09 (t, 4H, N-CH-C*H*-CH-C*H*-CH-N), 4.80 (t, 4H, N-C*H*_*2*_-CH_2_-C*H*_*2*_-N), 2.86-2.75 (m, 2H, N-CH_2_-C*H*_*2*_-CH_2_-N). ^13^C NMR (68 MHz, D_2_O): δ 146.54 (N-CH-CH-*C*H-CH-CH-N), 144.62 (N-*C*H-CH-CH-CH-*C*H-N), 128.85 (N-CH-*C*H-CH-*C*H-CH-N), 58.30 (N-*C*H_2_-CH_2_-*C*H_2_-N), 31.96 (N-CH_2_-*C*H_2_-CH_2_-N). MS m/z molecular ion: calcd. 100.0654; found 100.0672 (cation^+^).

**C**_**4**_ **(Pyr)**_**2**_ **Br**_**2**_**:** 1,4-bis(pyridinium-1-yl)butane dibromide. C_14_H_18_Br_2_N_2_; from 5.93 g (75 mmol) of pyridine and 6.48 g (30 mmol) of 1,4-dibromobutane, 10.22 g (27.32 mmol) of C_4_ (Pyr)_2_ Br_2_ was obtained (yield: 91%); ^1^H NMR (270 MHz, D_2_O): δ 8.85 (d, 4H, N-C*H*-CH), 8.54 (t, 2H, N-CH-CH-C*H*-CH-CH-N), 8.07 (t, 4H, N-CH-C*H*-CH-C*H*-CH-N), 4.72-4.61 (m, 4H, N-C*H*_*2*_-C_2_H_4_-C*H*_*2*_-N), 2.18-2.06 (m, 4H, N-CH_2_-C_2_*H*_*4*_-CH_2_-N). ^13^C NMR (68 MHz, D_2_O): δ 146.15 (N-CH-CH-*C*H-CH-CH-N), 144.45 (N-*C*H-CH-CH-CH-*C*H-N), 128.65 (N-CH-*C*H-CH-*C*H-CH-N), 61.03 (N-*C*H_2_-C_2_H_4_-*C*H_2_-N), 27.49 (N-CH_2_-*C*_*2*_H_4_-CH_2_-N). MS m/z molecular ion: calcd. 107.0732; found 107.0696 (cation^+^).

**C**_**6**_ **(Pyr)**_**2**_ **Br**_**2**_**:** 1,6-bis(pyridinium-1-yl)hexane dibromide. C_16_H_22_Br_2_N_2_; from 5.93 g (75 mmol) of pyridine and 7.32 g (30 mmol) of 1,6-dibromohexane, 11.55 g (28.72 mmol) of C_6_ (Pyr)_2_ Br_2_ was obtained (yield: 96%); ^1^H NMR (270 MHz, D_2_O): δ 8.83 (d, 4H, N-C*H*-CH), 8.53 (t, 2H, N-CH-CH-C*H*-CH-CH-N), 8.05 (t, 4H, N-CH-C*H*-CH-C*H*-CH-N), 4.60 (t, 4H, N-C*H*_*2*_-C_4_H_8_-C*H*_*2*_-N), 2.06-1.94 (m, 4H, N-CH_2_-C*H*_*2*_-C_2_H_4_-C*H*_*2*_-CH_2_-N), 1.44-1.33 (m, 4H, N-C_2_H_4_-C_2_*H*_*4*_-C_2_H_4_-N). ^13^C NMR (68 MHz, D_2_O): δ 145.79 (N-CH-CH-*C*H-CH-CH-N), 144.39 (N-*C*H-CH-CH-CH-*C*H-N), 128.45 (N-CH-*C*H-CH-*C*H-CH-N), 61.92 (N-*C*H_2_-C_4_H_8_-*C*H_2_-N), 30.49 (N-CH_2_-*C*H_2_-C_2_H_4_-*C*H_2_-CH_2_-N), 24.99 (N-C_2_H_4_-*C*_*2*_H_4_-C_2_H_4_-N). MS m/z molecular ion: calcd. 121.0888; found 121.0873 (cation^+^).

**C**_**8**_ **(Pyr)**_**2**_ **Br**_**2**_**:** 1,8-bis(pyridinium-1-yl)octane dibromide. C_18_H_26_Br_2_N_2_; from 5.93 g (75 mmol) of pyridine and 8.16 g (30 mmol) of 1,8-dibromooctane, 12.17 g (28.30 mmol) of C_8_ (Pyr)_2_ Br_2_ was obtained (yield: 94%); ^1^H NMR (270 MHz, D_2_O): δ 8.82 (d, 4H, N-C*H*-CH), 8.52 (t, 2H, N-CH-CH-C*H*-CH-CH-N), 8.04 (t, 4H, N-CH-C*H*-CH-C*H*-CH-N), 4.58 (t, 4H, N-C*H*_*2*_-C_6_H_12_-C*H*_*2*_-N), 2.04-1.93 (m, 4H, N-CH_2_-C*H*_*2*_-C_4_H_8_-C*H*_*2*_-CH_2_-N), 1.37-1.24 (m, 8H, N-CH_2_-CH_2_-C_4_*H*_*8*_-CH_2_-CH_2_-N). ^13^C NMR (68 MHz, D_2_O): δ 145.69 (N-CH-CH-*C*H-CH-CH-N), 144.37 (N-*C*H-CH-CH-CH-*C*H-N), 128.39 (N-CH-*C*H-CH-*C*H-CH-N), 62.12 (N-*C*H_2_-C_6_H_12_-*C*H_2_-N), 30.65 (N-CH_2_-*C*H_2_-C_4_H_8_-*C*H_2_-CH_2_-N), 28.00 (N-C_2_H_4_-*C*H_2_-C_2_H_4_-*C*H_2_-C_2_H_4_-N), 25.25 (N-C_3_H_6_-*C*_*2*_H_4_-C_3_H_6_-N). MS m/z molecular ion: calcd. 135.1044; found 135.1037 (cation^+^).

**C**_**12**_ **(Pyr)**_**2**_ **Br**_**2**_**:** 1,12-bis(pyridinium-1-yl)dodecane dibromide. C_22_H_34_Br_2_N_2_; from 5.93 g (75 mmol) of pyridine and 9.84 g (30 mmol) of 1,12-dibromododecane, 13.86 g (28.51 mmol) of C_12_ (Pyr)_2_ Br_2_ was obtained (yield: 95%); ^1^H NMR (270 MHz, D_2_O): δ 8.82 (d, 4H, N-C*H*-CH), 8.51 (t, 2H, N-CH-CH-C*H*-CH-CH-N), 8.04 (t, 4 H, N-CH-C*H*-CH-C*H*-CH-N), 4.58 (t, 4H, N-C*H*_*2*_-C_10_H_20_-C*H*_*2*_-N), 2.03-1.92 (m, 4H, N-CH_2_-C*H*_*2*_-C_8_H_16_-C*H*_*2*_-CH_2_-N), 1.30-1.19 (m, 16H, N-CH_2_-CH_2_-C_8_*H*_*16*_-CH_2_-CH_2_-N). ^13^C NMR (68 MHz, D_2_O): δ 145.68 (N-CH-CH-*C*H-CH-CH-N), 144.36 (N-*C*H-CH-CH-CH-*C*H-N), 128.38 (N-CH-*C*H-CH-*C*H-CH-N), 62.17 (N-*C*H_2_-C_10_H_20_-*C*H_2_-N), 30.69 (N-CH_2_-*C*H_2_-C_8_H_16_-*C*H_2_-CH_2_-N), 28.68 (N-C_2_H_4_-*C*H_2_-C_6_H_12_-*C*H_2_-C_2_H_4_-N), 28.59 (N-C_3_H_6_-*C*H_2_-C_4_H_8_-*C*H_2_-C_3_H_6_-N), 28.23 (N-C_4_H_8_-*C*H_2_-C_2_H_4_-*C*H_2_-C_4_H_8_-N), 25.35 (N-C_5_H_10_-*C*_*2*_H_4_-C_5_H_10_-N). MS m/z molecular ion: calcd. 163.1357; found 163.1370 (cation^+^).

**C**_**3**_ **(Pyr) (MIm) Br**_**2**_**:** 1-(pyridinium-1-yl)-3-(3-methylimidazolium-1-yl)propane dibromide C_12_H_17_Br_2_N_3_; from 4.42 g (15 mmol) of 1-(3-bromopropyl)pyridinium bromide and 1.48 g (18 mmol) of 1-methylimidazole, 5.1 g (14.04 mmol) of C_3_ (Pyr) (MIm) Br_2_ was obtained (yield: 94%); ^1^H NMR (270 MHz, methanol-d4): δ 9.18 (d, 2H, N-C*H*-CH-CH-CH-C*H*-N), 9.14 (s, 1H, N-C*H*-N) 8.66 (t, 1H, N-CH-CH-C*H*-CH-CH-N), 8.18 (t, 2H, N-CH-C*H*-CH-C*H*-CH-N), 7.78 (t, 1H, N-C*H*-CH-N), 7.64 (t, 1H, N-CH-C*H*-N), 4.86 (t, 32H, N-C*H*_*2*_-CH_2_-CH_2_-N), 4.50 (t, 2H, N-CH_2_-CH_2_-C*H*_*2*_-N), 3.98 (s, 3H, N-C*H*_*3*_), 2.77-2.66 (m, 2H, N-CH_2_-C*H*_*2*_-CH_2_-N). ^13^C NMR (68 MHz, methanol-d4): δ 147.48 (N-CH-CH-*C*H-CH-CH-N), 146.41 (N-*C*H-CH-CH-CH-*C*H-N), 138.48 (N-*C*H-N), 129.86 (N-CH-*C*H-CH-*C*H-CH-N), 125.40 (N-*C*H-CH-N), 123.89 (N-CH-*C*H-N), 59.68 (N-*C*H_2_-CH_2_-CH_2_-N), 47.61 (N-CH_2_-CH_2_-*C*H_2_-N), 36.95 (N-CH_3_), 32.81 (N-CH_2_-*C*H_2_-CH_2_-N). MS m/z molecular ion: calcd. 101.5708; found 101.5682 (cation^+^).

**C**_**3**_ **(Pyr) (MPyrr) Br**_**2**_**:** 1-(pyridinium-1-yl)-3-(1-methylpyrrolidinium-1-yl)propane dibromide C_13_H_22_Br_2_N_2_; from 4.42 g (15 mmol) of 1-(3-bromopropyl)pyridinium bromide and 1.66 g (19.5 mmol) of 1-methylpyrrolidine, 4.71 g (12.86 mmol) of C_3_ (Pyr) (MPyrr) Br_2_ was obtained (yield: 86%); ^1^H NMR (270 MHz, methanol-d4): δ 9.22 (d, 2H, N-C*H*-CH-CH-CH-C*H*-N), 8.67 (t, 1H, N-CH-CH-C*H*-CH-CH-N), 8.20 (t, 2H, N-CH-C*H*-CH-C*H*-CH-N), 4.85 (t, 2H, N_aromatic_-C*H*_*2*_-CH_2_-CH_2_-N), 3.71-3.63 (m, 6H, N_aromatic_-CH_2_-CH_2_-C*H*_*2*_-N y C*H*_*2*_-N_pyrrol_-C*H*_*2*_), 3.17 (s, 3H, N-C*H*_*3*_), 2.70-2.64 (m, 2H, N_aromatic_-CH_2_-C*H*_*2*_-CH_2_-N), 2.31-2.24 (m, 4H, N-CH_2_-C*H*_*2*_-C*H*_*2*_-CH_2_-N). ^13^C NMR (68 MHz, methanol-d4): δ 147.58 (N-CH-CH-*C*H-CH-CH-N), 146.45 (N-*C*H-CH-CH-CH-*C*H-N), 129.87 (N-CH-*C*H-CH-*C*H-CH-N), 66.20y 66.16 (*C*H_2_-N_pyrrol_-*C*H_2_), 61.53 (N_aromatic_-CH_2_-CH_2_-*C*H_2_-N), 59.46 (N_aromatic_-*C*H_2_-CH_2_-CH_2_-N), 49.60 (N-*C*H_3_), 27.30 (N-CH_2_-*C*H_2_-*C*H_2_-CH_2_-N), 22.83 (N_aromatic_-CH_2_-*C*H_2_-CH_2_-N). MS m/z molecular ion: calcd. 103.0888; found 103.0862 (cation^+^).

Melting point temperature of the ILs or molten salts which are solid at room temperature was recorded on a Büchi Melting Point B-540 instrument. When the thermal decomposition of the IL or the molten salt was reached before its melting, thermal decomposition temperature was determined by Differential Scanning Calorimetry on a DSC 2920 (TA instruments) instrument.

## References

[1] Montalbán M.G., Víllora G., Licence P. (2018). Ecotoxicity assessment of dicationic versus monocationic ionic liquids as a more environmentally friendly alternative. Ecotoxicol. Environ. Safe.

[2] Sadeghi R., Shekaari H., Hosseini R. (2009). Effect of alkyl chain length and temperature on the thermodynamic properties of ionic liquids 1-alkyl-3-methylimidazolium bromide in aqueous and non-aqueous solutions at different temperatures. J. Chem. Thermodyn..

[3] Shekaari H., Kazempour A. (2011). Effect of ionic liquid, 1-octyl-3-methylimidazolium bromide on the thermophysical properties of aqueous D-glucose solutions at 298.15K. Fluid Phase Equilib..

[4] Dzyuba S.V., Bartsch R.A. (2001). Efficient synthesis of 1-alkyl(aralkyl)-3-methyl(ethyl)imidazolium halides: precursors for room-temperature ionic liquids. J. Heterocycl. Chem..

[5] Papaiconomou N., Salminen J., Lee J.-M., Prausnitz J.M. (2007). Physicochemical properties of hydrophobic ionic liquids containing 1-octylpyridinium, 1-octyl-2-methylpyridinium, or 1-octyl-4-methylpyridinium cations. J. Chem. Eng. Data.

[6] Goossens K., Lava K., Nockemann P., Van Hecke K., Van Meervelt L., Driesen K., Gçrller-Walrand C., Binnemans K., Cardinaels T. (2009). Pyrrolidinium ionic liquid crystals. Chem. Eur. J.

[7] Takanobu I., Hiroshi I., Shogo K., Yanagimachi T. (1952). Syntheses of several N-alkyl pyridinium hydroxides and their actions on mice. Eisei Shikensho Hokoku.

[8] Deyko A., Lovelock K.R.J., Corfield J.-A., Taylor A.W., Gooden P.N., Villar-Garcia I.J., Licence P., Jones R.G., Krasovskiy V.G., Chernikova E.A., Kustov L.M. (2009). Measuring and predicting Δ_vap_H_298_ values of ionic liquids. Phys. Chem. Chem. Phys..

[9] Men S., Lovelock K.R.J., Licence P., Cremer T., Paape N., Wasserscheid P., Fröba A.P., Maier F., Steinrück H.P., Kirchner B., Maier F., Steinrück H.P. (2011). X-ray photoelectron spectroscopy of pyrrolidinium-based ionic liquids: cation–anion interactions and a comparison to imidazolium-based analogues. Phys. Chem. Chem. Phys..

[10] Dzyuba S.V., Bartsch R.A. (2002). Influence of structural variations in 1-Alkyl(aralkyl)-3-methylimidazolium hexafluorophosphates and bis(trifluoromethylsulfonyl)imides on physical properties of the ionic liquids. ChemPhysChem.

[11] Hardacre C., Holbrey J.D., Katdare S.P., Seddon K.R. (2002). Alternating copolymerisation of styrene and carbon monoxide in ionic liquids. Green Chem..

[12] Longo L.S., Smith E.F., Licence P. (2016). Study of the stability of 1-alkyl-3-methylimidazolium hexafluoroantimonate(V) based ionic liquids using X-ray photoelectron spectroscopy. ACS Sustain. Chem. Eng..

[13] Xing X., Zhao G., Cui J., Zhang S. (2012). Isobutane alkylation using acidic ionic liquid catalysts. Catal. Commun..

[14] Tadesse H., Blake A.J., Champness N.R., Warren J.E., Rizkallah P.J., Licence P. (2012). Supramolecular architectures of symmetrical dicationic ionic liquid based systems. CrystEng. Comm..

[15] Rao W., Mitchell D., Licence P., Barrett D.A. (2014). The use of dicationic ion-pairing compounds to enhance the ambient detection of surface lipids in positive ionization mode using desorption electrospray ionisation mass spectrometry. Rapid Commun. Mass Spectrom..

[16] Lee H.M., Lu C.Y., Chen C.Y., Chen W.L., Lin H.C., Chiu P.L., Cheng P.Y. (2004). Palladium complexes with ethylene-bridged bis(N-heterocyclic carbene) for C–C coupling reactions. Tetrahedron.

[17] Lee M., Niu Z., Slebodnick C., Gibson H.W. (2010). Structure and properties of N,N-Alkylene Bis(N′-Alkylimidazolium) salts. J. Phys. Chem. B.

[18] Anderson J.L., Ding R., Ellern A., Armstrong D.W. (2005). Structure and properties of high stability geminal dicationic ionic liquids. J. Am. Chem. Soc..

[19] Lostun D., Perez C.J., Licence P., Barrett D.A., Ifa D.R. (2015). Reactive DESI-MS imaging of biological tissues with dicationic ion-pairing compounds. Anal. Chem..

[20] Chang J.-C., Ho W.-Y., Sun I.-W., Chou Y.-K., Hsieh H.-H., Wu T.-Y., Liang S.-S. (2010). Synthesis and properties of new (μ-oxo)bis[trichloroferrate(III)] dianion salts incorporated with dicationic moiety. Polyhedron.

